# Analgesic exposure in pregnant rats affects fetal germ cell development with inter-generational reproductive consequences

**DOI:** 10.1038/srep19789

**Published:** 2016-01-27

**Authors:** Afshan Dean, Sander van den Driesche, Yili Wang, Chris McKinnell, Sheila Macpherson, Sharon L. Eddie, Hazel Kinnell, Pablo Hurtado-Gonzalez, Tom J. Chambers, Kerrie Stevenson, Elke Wolfinger, Lenka Hrabalkova, Ana Calarrao, Rosey AL Bayne, Casper P. Hagen, Rod T. Mitchell, Richard A. Anderson, Richard M. Sharpe

**Affiliations:** 1 MRC Centre for Reproductive Health, The Queen’s Medical Research Institute University of Edinburgh, 47 Little France Crescent, Edinburgh EH16 4TJ, UK

## Abstract

Analgesics which affect prostaglandin (PG) pathways are used by most pregnant women. As germ cells (GC) undergo developmental and epigenetic changes in fetal life and are PG targets, we investigated if exposure of pregnant rats to analgesics (indomethacin or acetaminophen) affected GC development and reproductive function in resulting offspring (F1) or in the F2 generation. Exposure to either analgesic reduced F1 fetal GC number in both sexes and altered the tempo of fetal GC development sex-dependently, with delayed meiotic entry in oogonia but accelerated GC differentiation in males. These effects persisted in adult F1 females as reduced ovarian and litter size, whereas F1 males recovered normal GC numbers and fertility by adulthood. F2 offspring deriving from an analgesic-exposed F1 parent also exhibited sex-specific changes. F2 males exhibited normal reproductive development whereas F2 females had smaller ovaries and reduced follicle numbers during puberty/adulthood; as similar changes were found for F2 offspring of analgesic-exposed F1 fathers or mothers, we interpret this as potentially indicating an analgesic-induced change to GC in F1. Assuming our results are translatable to humans, they raise concerns that analgesic use in pregnancy could potentially affect fertility of resulting daughters and grand-daughters.

An altered environment during fetal development can affect health in offspring, and can contribute to disorders of growing prevalence in modern societies, such as obesity and cardiometabolic disorders^1^,^2^. These changes, which may represent ‘side effects’ of adaptational changes by the fetus in response to environmental/growth cues during development^3^, also extend to altered reproductive development and/or function^4^,^5^,^6^. Furthermore, if the developing gonads of the fetus are adversely affected by environmental factors, changes in the germ cells could lead to alterations in the next (F2) generation. There is growing experimental and human evidence for such intergenerational effects^3^,^7^,^8^, raising the possibility that lifestyle, diet and exposures prior to and during human pregnancy can impact the wellbeing and health of subsequent generations (children and grandchildren).

In recent years, considerable attention has focussed on the possibility that fetal exposure to ‘endocrine disruptors’ - weakly endocrine-active environmental chemicals - might cause adverse effects in the fetus^4^. In contrast, potential fetal effects resulting from exposure to pharmaceutical compounds have received comparatively little attention, although a recent study^9^ showed effects of several analgesics on hormone production by the human fetal testis. Of significant interest is the analgesic/anti-pyretic acetaminophen (paracetamol), which is used by the majority of women during pregnancy^10^,^11^. It readily crosses the placenta into the fetus^12^ but is generally recommended as safe for use in pregnancy (eg in UK: http://www.nhs.uk/chq/Pages/2397.aspx). Moreover, acetaminophen is available over-the-counter (OTC), and is incorporated into a range of OTC medicines. Many of these issues apply also to non-steroidal anti-inflammatory compounds (NSAIDs) such as ibuprofen, aspirin and indomethacin, as some of these are also available OTC and are widely used in pregnancy, even if they are not recommended for such use other than on medical advice^13^.

Pregnancy exposure to acetaminophen has recently been associated with increased risk of neurodevelopmental^14^ and ADHD-type disorders^15^ in children, and four independent studies have shown an increased risk of cryptorchidism in boys born to mothers who reported protracted use of acetaminophen alone, or with NSAIDs, during late 1^st^ and early 2^nd^ trimester of pregnancy^16^,^17^,^18^,^19^. *In vitro* and *vivo* studies in rats^9^,^18^,^20^,^21^ or using human fetal testis xenografts^21^ found inhibitory effects of acetaminophen on testosterone production. There are no reports on altered germ cell number or function in either men or women but we have previously reported expression of the prostaglandin (PG) synthetic enzymes cyclooxygenase-2 (COX2) and PTGES and PG receptors in oocytes in the human fetal ovary, and that prostaglandins may be involved in human oocyte development prior to primordial follicle formation^22^. The present study shows that COX2 and PG receptors are also present in fetal germ and/or somatic cells of both male and female rats, suggesting that PGs might play a common and conserved role in fetal germ cell development. This observation prompted us to investigate the effects of analgesic exposure on germ cell development in male and female rats. Our results show that fetal germ cell development in either sex is affected by analgesic exposure and this results in reproductive changes, not only in exposed individuals, but also in F2 progeny which they later give rise to.

## Results

### Fetal germ and somatic cells as sources and targets for PGs in the rat

**Figure 1:**
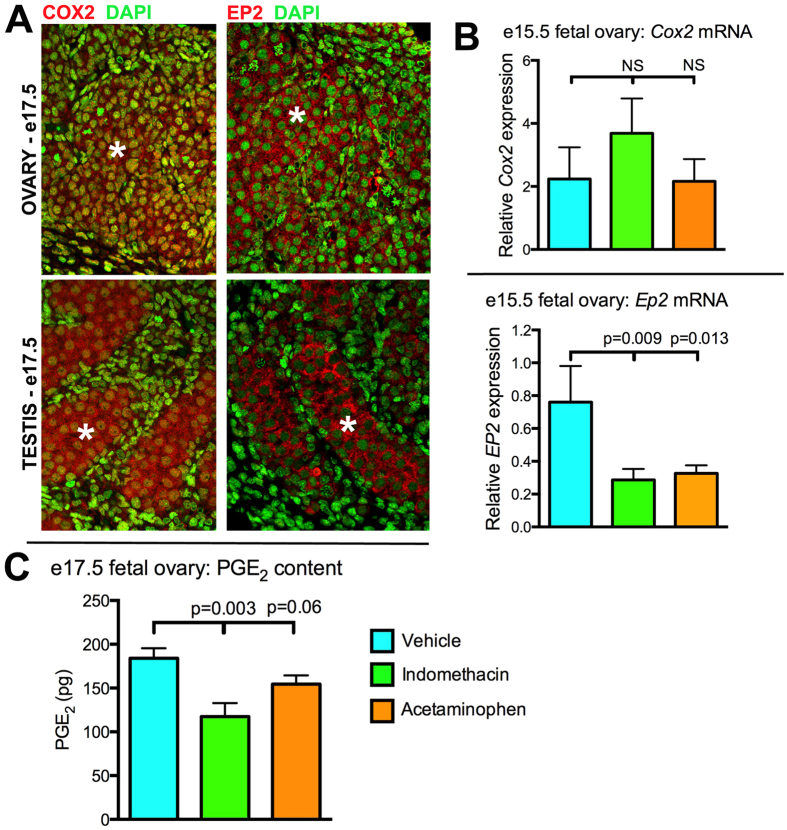
The fetal rat gonads as a source and target for prostaglandins (PGs). (**A**) Immunoexpression of cyclo-oxygenase-2 (COX2) and PGE_2_ receptors (EP2) in germ cells (asterisks) of the fetal (e17.5) rat ovary and testis. (**B**) The effect of exposure to indomethacin or acetaminophen on *Cox2* and *Ep2* mRNA expression at e15.5 in the F1 fetal ovary (Values are Means ± SEM for n = 6–9). (**C**) Effect of analgesic exposure on F1 fetal rat ovarian PGE_2_ content 3h after a single administration of vehicle or analgesic on e17.5 (Means ± SEM for n = 5).

Fetal gonads in both sexes expressed COX2 and the germ cells are PG targets i.e they express PG receptors, for example EP2 (Fig. 1A). This suggested that analgesic exposure in pregnancy might perturb the physiological processes regulated by PGs in fetal germ cells. This possibility was reinforced by our finding that fetal ovarian PGE_2_ levels (Fig. 1C) and/or *Ep2* mRNA expression (Fig. 1B) were reduced by maternal analgesic exposure, whereas *Cox2* mRNA expression was unaffected (Fig. 1B); we have previously shown a similar effect of indomethacin on PGE_2_ levels in the fetal rat testis^23^.

As germ cells undergo important changes in fetal life involving sex-specific differentiation and epigenetic reprogramming^24^,^25^,^26^, we considered the possibility that analgesic exposure might affect these events in the F1 generation and potentially lead to altered germ cell development/function in resulting F2 offspring. For these studies we used the same dose of indomethacin (0.8 mg/kg) used in earlier rat studies on steroidogenesis^23^. For acetaminophen, we used a single high, but non-toxic, daily dose of 350 mg/kg/day, based on previous rat studies^18^,^20^,^21^. For comparison with human exposure we measured plasma acetaminophen 1h after administration of 350 mg/kg/day, which resulted in levels of 49.4 ± 13.9 μg/ml (means ± SEM, N = 4); plasma acetaminophen was undetectable in 4 controls. In humans, a therapeutic dose of acetaminophen results in blood levels ranging from 6–20.8 μg/ml at ~1 h after oral administration, including in pregnant women^27^,^28^. Acetaminophen (350 mg/kg) administration to pregnant rats had no obvious toxic effect, and fetal bodyweight of both male and female offspring at e21.5 was marginally increased (by 2.2–5.3%) compared with controls (Supplementary Fig. 1). In contrast, fetal bodyweight of male and female offspring from indomethacin-exposed mothers at e21.5 was significantly reduced (by 9.3–10.8%) compared with controls (Supplementary Fig. 1).

### Maternal analgesic exposure affects fetal germ cells in both female and male rat fetuses

**Figure 2:**
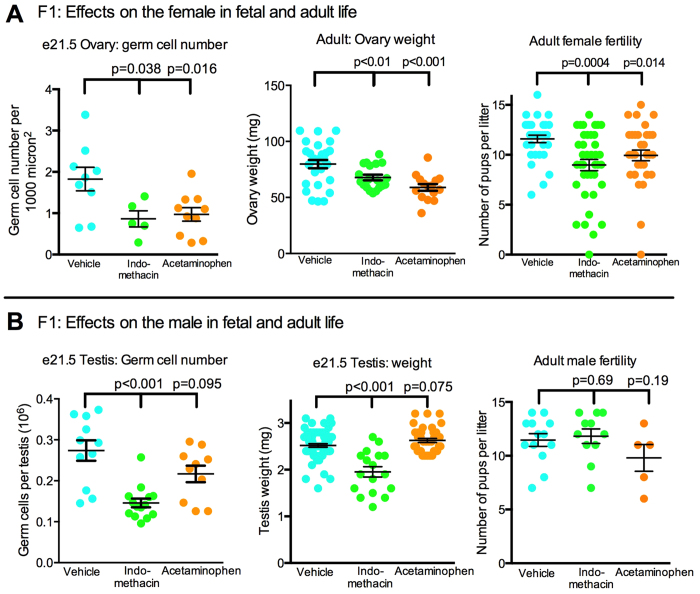
Effect of fetal exposure to analgesics on (top) ovarian development and fertility in F1 female rats, and (bottom) corresponding changes in F1 males. (**A**) Germ cell number at e21.5 (n = 5–10 animals per group), adult ovarian weight (n = 15–27 animals per group) and fertility after mating with normal untreated stud males (n = 30–36 animals per group). (**B**) Germ cell number at e21.5 in the testis (n = 10–14 animals per group), together with testis weight at the same age (n = 17–64 animals per group) and fertility after mating with normal untreated adult females (n = 5–13 animals per group). Black horizontal bars show Means ± SEM. Note that controls used for indomethacin and acetaminophen studies were pooled for analysis as they did not differ significantly for any of the measured parameters. In each group, animals were from 4–11 different litters.

Intra-uterine analgesic exposure (indomethacin or acetaminophen) reduced the number of germ cells in the fetal ovary and reduced fetal testis weight and the number of germ cells in males (indomethacin only) (Fig. 2). The analgesic-induced reduction in fetal ovarian germ cell number was of particular concern, as the lifetime complement of oocytes is formed in utero at/around the time of birth in women and rodents: this reduction could therefore impact oocyte number in adult life. Consistent with this, we found significantly reduced ovarian size and female fertility (number of pups per litter) in adulthood in females exposed in utero to either indomethacin or acetaminophen (Fig. 2). In contrast, in males exposed in utero to indomethacin, the reduction in fetal germ cell number evident at e21.5 (Fig. 2), was compensated for by early puberty (Supplementary Fig. 2) and adult testis size (not shown) and male fertility (number of pups per litter; Fig. 2) was comparable to controls.

### Effects of fetal analgesic exposure on germ cell development

**Figure 3:**
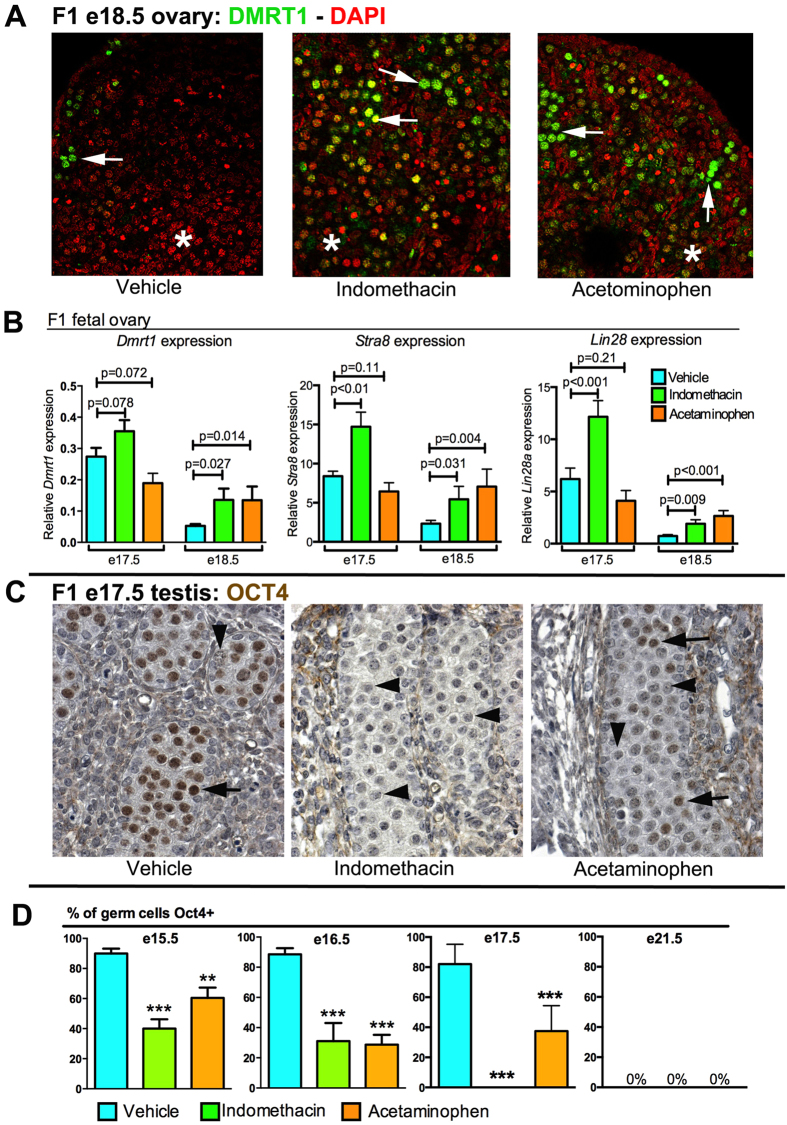
Effect of fetal analgesic exposure on the tempo of fetal germ cell development in the F1 fetal ovary (**A**,**B**) and testis (**C**,**D**). (**A**) Representative immunoexpression results for DMRT1 (green) in e18.5 ovaries from fetuses exposed to vehicle or to analgesic, highlighting regional differences in the proportion of oocytes still expressing DMRT1 (green) and those in which DMRT1 has been switched off (asterisks). (**B**) Evidence for delayed oocyte development in analgesic-exposed fetuses based on the temporal change in expression of *Dmrt1*, *Stra8* and *Lin28* mRNA expression at e17.5 and e18.5 (Means ± SEM for 9–18 animals per group from 3–5 different litters). (**C**) Germ cell-specific nuclear expression of OCT4 (brown; black arrows) in the fetal testis was reduced substantially by exposure to analgesic with all (indomethacin) or most (acetaminophen) germ cells prematurely losing expression of OCT4 by e17.5, unlike in controls. (**D**) The proportion of germ cells expressing OCT4 was quantified at e15.5, e16.5, e17.5 and e21.5 for 5–6 animals per group at each age from a minimum of 5 litters (Means ± SEM). **p < 0.01, ***p < 0.001, in comparison with respective control group.

In the fetal ovary, the developmental pathway for germ cells is to switch off pluripotency factors and enter meiosis. We monitored meiosis in F1 female fetuses using immunoexpression of the mitosis-meiosis regulator DMRT1, which is germ cell-specific after e15.5 in the rat (Supplementary Fig. 3 and Refs^29^,^30^,^31^); thus germ cell loss of DMRT1 expression can be used as an index of completion of meiotic entry. In control females between e15.5 and e17.5 most germ cells exhibited nuclear expression of DMRT1. At e18.5, germ cells that were yet to enter or still initiating meiosis showed translocation of DMRT1 from the nucleus to the cytoplasm, whereas those that had completed meiotic entry lacked DMRT1 expression completely (Supplementary Fig. 3). However, there was considerable variation in the proportion of DMRT1^+^ oocytes within e18.5 ovaries (Fig. 3A), making quantification of changes in germ cell development based on immunoexpression difficult. Instead, at e17.5–e18.5 we determined whole ovarian mRNA expression of two markers of meiotic entry (*Dmrt1* and *Stra8)* and a pluripotency marker (*Lin28*), expression of which is lost in ovarian germ cells in rats and humans prior to meiotic entry^32^,^33^. All three markers exhibited a substantial (p < 0.001) reduction in expression from e17.5 to e18.5 in controls (Fig. 3B). However, this decrease was significantly attenuated at e18.5 in ovaries from indomethacin- and acetaminophen-exposed animals (Fig. 3B), suggesting a delay in normal germ cell development and entry into meiosis in females.

Male fetal germ cells do not enter meiosis but undergo a differentiation step when they switch off pluripotency markers (eg OCT4) and simultaneously cease proliferation, a process commencing at ~e15.5 and complete by e19.5 in rats^34^. We used germ cell OCT4 immunoexpression to monitor this differentiation. Unexpectedly, this revealed that analgesic exposure significantly accelerated the loss of OCT4 in germ cells between e15.5 and e17.5, in comparison with age-matched controls (Fig. 3C,D). Thus analgesic exposure caused advanced differentiation of fetal germ cell development in males, an opposite effect to that seen in females.

### Effect of fetal analgesic exposure (F1 animals) on F2 offspring; inter-generational effects

As fetal germ cells in the F1 generation are the source of the next (F2) generation, we undertook a pilot investigation (Experiment 1) to investigate whether exposure of fetuses to indomethacin had any gross reproductive developmental consequence for F2 animals derived from these F1 parents. Thus, 4 indomethacin-exposed F1 females and 4 F1 males (from 4 different litters) were mated in adulthood with untreated control animals and the resulting F2 offspring sampled in adulthood (n = 31–43 offspring/group). Corresponding matings of vehicle-exposed F1 females and males (n = 4 of each) with untreated controls generated F2 adult control offspring (n = 49); as F2 control data was unaffected by the sex of the F1 parent used to generate F2 offspring, data per sex were pooled for comparison with analgesic treatment groups.

**Figure 4:**
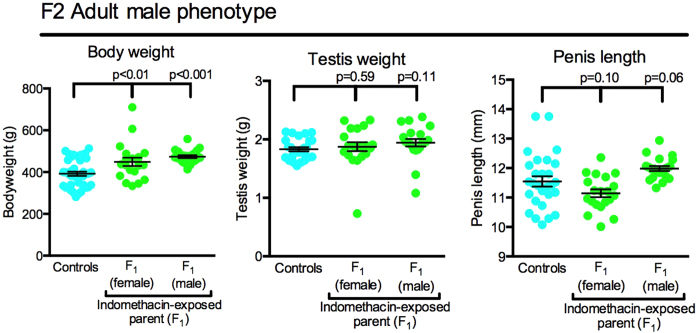
Effect of fetal exposure of the F1 generation to indomethacin on bodyweight and gross reproductive phenotype of second generation (F2) adult males. Black horizontal bars show Means ± SEM. Mated F1 animals derived from 7 separate litters whilst data for F2 animals derived from 5–6 litters.

**Figure 5:**
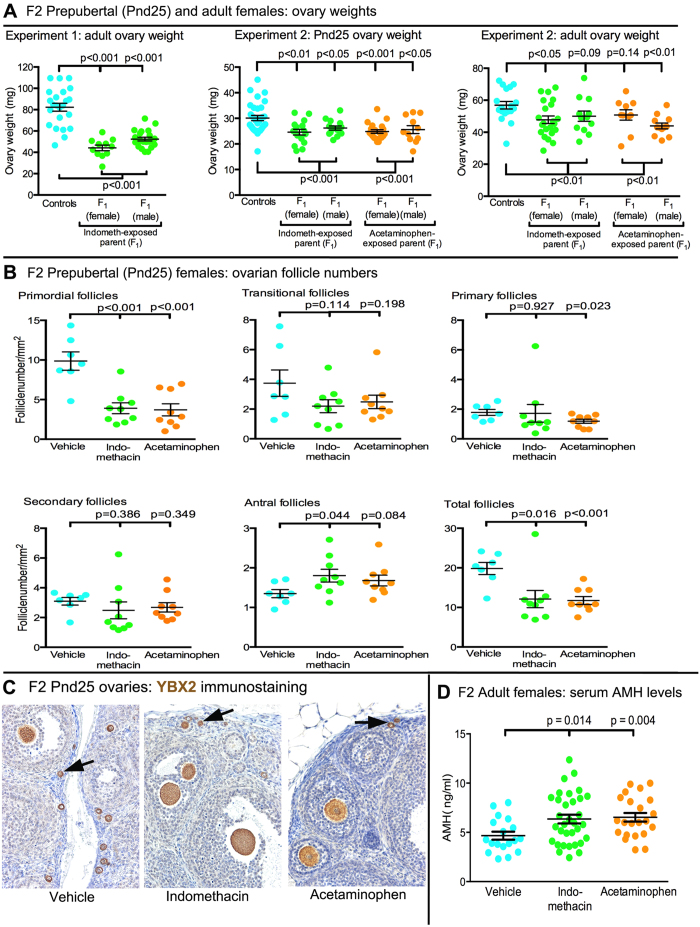
Effect of fetal exposure of the F1 generation to analgesics on ovarian size and function in second generation (F2) females. A Ovarian weights at postnatal day 25 (Pnd25) or adulthood were reduced overall in F2 females of analgesic-exposed parents, an effect that was generally evident irrespective of which F1 parent had been exposed to analgesic in utero. B Follicle classification and counts in the ovaries of representative prepubertal females from experiment 2 (panel A) based on immunostaining of oocytes for YBX2 (panel C). Follicle counts revealed a highly significant decrease in primordial follicle number (arrowed in panel C) in Pnd25 females derived from an F1 parent exposed in utero to analgesic and there was a trend towards increased numbers of antral follicles in the same ovaries (data derived from equal numbers of paternal and maternal ‘treatment exposed’ parents). D Serum AMH levels in adult F2 females (N=18–33 per group). Black horizontal bars show Means ± SEM. Mated F1 animals derived from 7 separate litters whilst data for F2 animals derived from 5-6 litters.

F2 adult males whose F1 parent was exposed in utero to indomethacin were significantly heavier than corresponding controls but the gross reproductive phenotype was broadly normal based on testis size and penis length (Fig. 4). In contrast, F2 adult females, derived from indomethacin-exposed mothers or fathers exhibited a significant (p < 0.001) decrease (36–46%) in ovarian weight (Fig. 5A). As the latter change could be indicative of reduced oocyte number (ovarian reserve), we repeated the experiment (Experiment 2) and investigated F2 female offspring at e21.5, prepuberty (Postnatal day 25; Pnd25) and adulthood after mating F1 parents (4–6 per treatment group from 4 litters) that had been exposed in utero to vehicle, indomethacin or acetaminophen. This study confirmed the significant reduction in ovarian weight in F2 offspring of analgesic-exposed parents seen in Experiment 1 at both time points, a 13–18% reduction at Pnd25 and 11–23% reduction in adulthood (Fig. 5A). This reduction was equally evident in F2 offspring of indomethacin- and acetaminophen-exposed parents and was independent of the sex of the analgesic-exposed F1 parent, as found in Experiment 1.

In F2 fetal ovaries at the end of gestation (e21.5), germ cell number was unchanged in animals derived from mating of an analgesic-exposed F1 parent (Supplementary Fig. 4). Therefore, F2 ovaries from pubertal (Pnd25) animals (7–8 per treatment group) were serially sectioned and the different follicle types counted after staining oocyte nuclei with YBX2 (Fig. 5C). This revealed a ~60% reduction in primordial follicle numbers in F2 offspring of analgesic-exposed parents (Fig. 5B), which was independent of the sex of the exposed F1 parent (data not shown). Overall, follicle numbers were reduced by ~40% in F2 female offspring of analgesic-exposed parents (Fig. 5B); however, this analysis also indicated increased (25–34%) numbers of antral follicles (Fig. 5B). To investigate this further, we measured serum anti-müllerian hormone (AMH) in F2 adult females, as AMH is produced primarily by large preantral/small antral follicles^35^. Serum AMH levels were significantly increased in F2 offspring of analgesic-exposed F1 parents (Fig. 5D).

## Discussion

Our results show for the first time that in utero exposure of rats to the analgesics indomethacin or acetaminophen, both of which target PG pathways, alters fetal germ cell number and development in both male and female fetuses. This results in modest but detrimental effects on F1 female, but not F1 male, fertility in adulthood. In normal females, germ cells undergo developmental changes in fetal life which culminate in their entry into and arrest in meiosis I, and primordial follicle formation. Failures or delays in this process can result in germ cell loss during late gestation^36^,^37^. Our demonstration of delayed development and meiotic entry of fetal germ cells after in utero exposure of female rat fetuses to analgesics may thus explain the reduced number of germ cells at the end of gestation in analgesic-exposed animals. This reduction in fetal oocyte number may account for the reduction in ovary weight and reduced litter size in adulthood in analgesic-exposed F1 females. Such effects are of concern because, in a human context, they might translate into reduced ovarian reserve and fertility and shortened reproductive lifespan, especially considering the high prevalence of analgesic use in pregnancy^10^,^11^.

Our findings raise added concern because fetal (F1) exposure of rats to either analgesic resulted in an effect in the second generation (F2 grand-daughters) that manifested as reduced ovarian size and markedly reduced follicle number in females but with evidence of increased follicle activation, reflected in numbers of antral follicles and serum AMH. The impact on F2 fertility (which was not studied) is unclear, but this pattern of effect on follicle development is similar to several mouse models of dysregulated primordial follicle arrest and activation^38^. As the majority of women use analgesics during pregnancy^10^,^11^, effects on their daughters and grand-daughters are possible, if the effects we show in the rat are translatable to the human. Our results are also in keeping with a recent study which showed in rats that a low-protein grandmaternal diet was able to alter ovarian reserves and antioxidant defence mechanisms in both daughters and grand-daughters^39^.

In contrast to the effects on fetal ovarian germ cells, in utero exposure of F1 males to analgesic significantly advanced germ cell development with early loss of pluripotency (OCT4). The mechanism for this loss was not explored, but in a variety of systems there is abundant evidence for epigenetic regulation of OCT4 expression^40^,^41^,^42^. As loss of pluripotency is tied with cessation of proliferation in fetal germ cells in the male^34^, advanced loss of OCT4 may explain the reduced fetal germ cell number in indomethacin-exposed males found at e21.5. However, this reduction was compensated for postnatally, which is another point of contrast with the F1 females, and a difference that readily explains the sex difference in effect on adult fertility after fetal analgesic exposure.

Our results suggest for the first time that PGs play hitherto unknown roles in fetal germ cell development in both sexes, but especially in the ovary. This is consistent with COX2 and PGE_2_ receptor expression in fetal germ cells (ref^22^ and present study) and with *Cox2* knockout mice showing multiple female reproductive deficiencies^43^. In adult animals there is abundant evidence for critical roles of PGs in gonadal function and germ cell development in both sexes^44^,^45^. Indomethacin and acetaminophen can both inhibit PG synthesis/action^46^,^47^,^48^, and such effects have been shown for fetal rat gonads (this study and refs^18^,^20^,^23^), although acetaminophen has additional effects^46^,^47^. As COX2 and PGE_2_ receptors are expressed in fetal somatic/germ cells in the human ovary, and PG alters ovarian *BDNF* expression which has effects on oocyte development/survival^22^, our results have clear human relevance.

A crucial question arising from our studies is the mechanism(s) via which fetal (F1) exposure to analgesics is able to alter ovarian development in the next generation (F2 grand-daughters). The present studies do not address this, but evidence from the literature points to various possibilities that we have considered, such as altered uterine development in F1 females^49^, behavioural changes in F1 parents^50^, or epigenetic changes to the germline^51^. As the analgesic effects on F2 ovaries were transmitted via both paternal and maternal F1 lines, we ruled out the possibility that the intergenerational effects could be due to a uterine effect in F1 females. Because there were differential effects of in utero analgesic exposure on fetal weights of F1 offspring (decreased with indomethacin, unchanged or increased with acetaminophen) we consider that these changes are also unlikely to account for the main findings of our studies. We did not evaluate behavioural changes in F1 parents, but any such adult behaviour changes would need to have been changed by in utero analgesic exposure of both fathers and mothers in a way that would lead to comparable effects on ovaries of F2 grand-daughters, which seems unlikely.

In contrast, there are several pieces of evidence from the literature that would support the possibility that fetal analgesic exposure might cause epigenetic changes to the F1 germ cells that then resulted in altered ovarian function in F2 females. First, our analgesic exposure regimen coincided with the period of chromatin/epigenetic remodelling of the (F1) fetal germ cells in both sexes^24^,^25^,^26^, events which also occur in the human in the first trimester of pregnancy^52^. Second, there are numerous examples of PGE_2_ modulation of DNA methylation in various cancers via effects on DNA methyltransferases^53^,^54^. Third, a recent study has shown that in human endometriotic cells experimental blockade of PGE_2_ action resulted in altered expression of most of the major components of the epigenetic regulatory machinery^55^; evaluation of whether there are comparable roles for PGE_2_ in the fetal gonads would seem an important avenue for future research.

Our study has limitations. The dosing regimens for indomethacin and acetaminophen differed, the former being administered from e15.5–e18.5 whilst acetaminophen was administered from e13.5–e21.5, stemming from different original purposes of studies using these compounds^18^,^21^,^23^. However, the similarity in effect of indomethacin and acetaminophen on germ cell development, irrespective of fetal sex, points to exposure prior to e18.5 as being critical as the analgesic effects on F1 germ cell differentiation that we describe all occurred prior to e18.5 in both sexes.

A limitation of the human health relevance of the present studies is that we administered only a single dose of analgesics, which may not match human exposure regimens. Although the indomethacin dose used (0.8 mg/kg/day) is within the human therapeutic range, the dose of acetaminophen which we used, resulted in blood levels of acetaminophen 2.5- to 8-fold higher than the levels reported in humans after normal therapeutic dosing (~60 mg/kg/day, divided into 4 doses) during pregnancy^27^,^28^. Based on acetaminophen effects on fetal testis steroidogenesis the rat is notably less sensitive to acetaminophen than the human^21^, so it is possible that a similar difference might apply to germ cell effects.

In conclusion, these data indicate that analgesics, commonly taken by women during pregnancy, decrease germ cell number and ovarian size in F1 offspring in the rat, and ovarian size and follicle numbers in F2 females (grand-daughters). Assessment of the human relevance of these findings may be possible via prospective epidemiological studies using new markers of oocyte number/reserves, such as AMH^56^.

## Materials and Methods

### Animals and treatments

All aspects of animal housing, management and treatment conformed to UK home office guidelines and all experiments were conducted under their specific project licence approval (RMS - PPL 60/4564); all experimental protocols were approved by the University of Edinburgh animal welfare and ethical review body as part of the project licence application. Wistar rats were housed under standard conditions and had free access to tap water and soy-free diet (SDS; Dundee, Scotland). Time-matings were established by the presence of a vaginal plug, defined as embryonic day 0.5 (e0.5). Pregnant animals were treated with indomethacin (Sigma-Aldrich, UK) during the masculinization programming window (MPW; e15.5–e18.5), or with acetaminophen (paracetamol) (Sigma-Aldrich, UK) from e13.5–e21.5 (encompassing the MPW, but extended after the MPW to replicate earlier studies; ref 15,19). Indomethacin was administered to pregnant dams by subcutaneous injection in corn oil^23^. The indomethacin dose which we used (0.8 mg/kg/day) derived from earlier studies showing that higher doses, (1–2 mg/kg/day) as used in mice, induced unacceptable litter loss and/or maternal intra-gastric bleeding^23^; at the dose of 0.8 mg/kg/day there was no obvious adverse effect on the mothers who showed normal bodyweight gain during pregnancy. Acetaminophen was administered by gavage as a suspension in corn oil at a dose of 350 mg/kg, based on earlier studies^18^,^21^, and was without any evidence of overt maternal toxicity in our studies, based on maternal bodyweight gain during pregnancy (this study and ref^21^). Control dams were treated only with corn oil/vehicle daily in the appropriate time window, with the respective route of administration. Data for offspring collected in fetal life were from a minimum of 5 litters per treatment group and for postnatal studies from 4–11 litters.

### Tissue recovery and measurements

Control and treated dams were killed on e15.5, e16.5, e17.5, e18.5, e21.5 or allowed to give birth, and resulting offspring then killed on either postnatal day (Pnd) 25 (= early puberty) or 90 (= adults). For fetal studies, pregnant dams were killed by inhalation of CO_2_ followed by cervical dislocation, fetuses were removed, and placed in ice-cold PBS (Sigma-Aldrich). E21.5 fetuses were weighed and then decapitated. Gonads were microdissected from fetuses and either stored at –80 °C for PGE_2_ ELISA quantification or used for mRNA analysis as below. Postnatal animals were killed by inhalation of CO_2_ followed by cervical dislocation. Bodyweight was measured before dissection and weighing of the gonads. Tissue was fixed in Bouins for 2–6 h (depending on tissue size) before transfer to 70% ethanol and embedding in paraffin wax using an automatic tissue processor.

### Prostaglandin E_2_ measurement

PGE_2_ was measured in ovaries of e17.5 fetuses recovered from vehicle- and analgesic-treated dams 4h after treatment. Gonads from each animal were pooled and homogenised in 0.05mM Tris/HCL PH 7.4 and PGE_2_ levels determined using Detect X Prostaglandin E_2_ Enzyme Immunoassay kit, according to manufacturers instructions (Arbor Assays, Michigan, US). PGE_2_ levels were read using an optical microplate reader (Labsystems, MutiSKan Ex, UK) and results analysed using MasterPlex™ ReaderFit software (MiraiBio Group, Hitachi Ltd).

### Acetaminophen measurement

Acetaminophen (APAP) was extracted from plasma by liquid-liquid extraction using acidified HPLC grade methanol (Fisher Scientific, Loughborough, UK). Briefly, 10μL plasma was enriched with 10ng APAP-d4 (Santa Cruz Biotechnology Inc, Heidelberg, Germany) as internal standard and 0.8mL methanol (w/0.2% acetic acid, Sigma Aldrich, Gillingham, UK) was added, vortexed and incubated for 20 min on ice. After centrifugation (3000*g*, 10 min, 10 ^o^C), the supernatant was dried under nitrogen at 40 ^0^C, reconstituted in mobile phase (0.2 mL water:methanol 65:35 v/v) and re-centrifuged. Chromatographic separation used an Aria CTC autosampler and Allegros pump on an ACE Excel 2 SuperC18 column (150 × 3 mm; 2 μm) protected by a Kinetex KrudKatcher® (Phenomenex, UK) at 20 ^o^C and detected on a TSQ Quantum Discovery triple quadrupole mass spectrometer (MS; Thermo Fisher Scientific, UK). The mobile phase consisted of 0.1% formic acid (Sigma Aldrich) in aqueous (A) and 0.1% formic acid in methanol (B) at a flow rate of 0.3 mL/min. Gradient elution was achieved with a total run time of 9 minutes from 35% to 5% B. APAP eluted at 3.95 min. The MS was operated in positive ion electrospray mode (300 ^o^C, 3 kV). Transitions monitored were *m/z* 152→110 and *m/z* 156.1→114.1 for APAP and APAP-d4, respectively.

### Serum AMH measurement

Serum AMH levels were measured using a commercial ELISA kit (Ansh Labs, Webster, USA) following the manufacturer’s instructions, except that samples were diluted 5-fold rather than 10-fold prior to analysis. The within assay CV was <7% across the working range and the lower limit of detection was 0.15 ng/ml; all samples were run in a single assay.

### Immunohistochemistry

Immunohistochemistry and immunofluorescence used methods detailed previously^22^,^33^,^57^. Sections of 5 μm were mounted onto coated slides (BDH Chemicals, Poole, UK), dewaxed and rehydrated. All immunohistochemistry for these studies used antigen retrieval by pressure cooking slides for 5 min in 0.01 M citrate buffer (pH 6.0). Slides were then incubated in 3% (vol/vol) hydrogen peroxide in methanol to block endogenous peroxidase activity and washed in Tris-buffered saline [TBS: 0.05 m Tris, 0.85% NaCl (pH 7.4)]. Nonspecific binding sites were blocked by incubation with appropriate normal serum diluted 1:5 in TBS containing 5% BSA (Sigma) before addition of primary antibody and overnight incubation at 4 °C, followed by the appropriate secondary antibody the following morning. The antibodies used, their dilutions, and sources are listed in Supplementary Table 1. For colorimetric immunohistochemistry (OCT4, TRA98, YBX2; Supplementary Table 1) slides were incubated for 30 min with the appropriate biotin-conjugated secondary antibody at a dilution of 1:500. The biotinylated antibody was linked to horseradish peroxidase (HRP) by 30 min incubation with Streptavidin-HRP complex (Dako) diluted 1:1000 in TBS. Antibody localization was visualised by application of diaminobenzidine (liquid DAB; Dako) until staining in control sections was optimal, and the reaction stopped by immersing slides in distilled water. Slides were counterstained with hematoxylin, dehydrated, and mounted using Pertex (Cell Path, Hemel Hempstead, UK).

For immunofluorescence, primary (COX2, DMRT1, EP2, VASA) and secondary antibodies were diluted as optimised (Supplementary Table 1). Detection used secondary antibodies that were IgG conjugated to HRP (Dako), diluted 1:200 in normal serum. Tyramide Signal Amplification (TSA plus cyanine system; Perkin-Elmer Life Sciences, Boston, MA) diluted 1:50 in the buffer supplied and incubated for 10 min, was used for detection. Nuclear counterstain (DAPI; Sigma-Aldrich) was diluted 1:500 in TBS and incubated for 10 min. Slides were washed in TBS and mounted in aqueous mounting medium (Permafluor; Beckman Coulter, High Wycombe, UK). Each immunohistochemistry run included negative controls with replacement of the primary antibody by blocking serum; these all showed minimal background staining (data not shown). Sections from control and treatment groups were mounted on the same slide where possible, and each experiment used sections from 3–12 animals per group/age.

### Image capture

Nonfluorescent images were captured using a Provis microscope (Olympus Optical, London, UK) fitted with a DCS330 digital camera (Eastman Kodak, Rochester, NY). Fluorescent images were captured using an LSM 510 Axiovert 100M confocal microscope (Carl Zeiss Ltd., Welwyn Garden City, UK). Images were compiled using Photoshop 7.0 (Adobe Systems Inc., Mountain View, CA).

### Gene expression analysis

For quantitative analysis of gene expression by RT-PCR, total RNA was extracted from e15.5 (n = 6–9), e17.5 (n = 11–18) or e18.5 (n = 9–14) ovary samples from F1 fetuses from the different treatment groups (controls, indomethacin, acetaminophen; n = 3–5 litters per group) using the RNeasy Micro Kit with on-column DNase digestion (Qiagen, UK). Both ovaries from each fetus were separated from the mesonephros and pooled and considered as one sample. Random hexamer primed cDNA was prepared using the Applied Biosystems Taqman^TM^ RT kit (Applied Biosystems, CA). Quantitative real time PCR (qRT-PCR) was performed on the ABI Prism Sequence Detection System (Applied Biosystems). Expression of the genes listed in Supplementary Table 2 was determined using the Roche Universal Probe Library (Roche Applied Sciences, Burgess Hill, UK) using the primer sequences and probe numbers listed in the Table. Expression of each gene was corrected using a ribosomal 18S internal control (Applied Biosystems Cat no. 4308329). All samples were performed in duplicate and a relative comparison was made to adult testis control cDNA.

### Determination of fetal ovarian germ cells per unit area

F1 ovaries (e21.5) were serial sectioned from 3–6 animals from each treatment group. A minimum of three sections per ovary, representing ~25, 50 and 75% points through the tissue block, were immunostained for the germ cell marker Tra98 and the number of positive cells quantified using Image-J software (http://rsb.info.nih.gov/ij/), which also enabled the area of the ovary to be measured on the same piece of tissue. The number of germ cells per micron^2^ of ovary was then determined.

### Determination of ovarian follicles per unit area

F2 ovaries (Pnd25) were serial sectioned from 3–6 animals from each treatment group. Three sections per ovary, representing ~10, 30 and 50% points through the tissue block, were immunostained for the oocyte marker YBX2^58^ and the number of primordial, transitional, primary, secondary and antral follicles quantified using Image-Pro software (Media Cybernetics Inc, USA). Only follicles containing a YBX2-positive oocyte nucleus were counted. The software was also used to measure the area of the same sections and the number of follicles per micron^2^ of ovary was then determined.

### Determination of germ cell number and % immunopositive germ cells in fetal gonads

Testis sections were analysed using a Zeiss Axio-Imager microscope (Carl Zeiss Ltd., Welwyn Garden City, UK) fitted with a Hitachi HV-C20 camera (Hitachi Denshi Europe, Leeds, UK) and a Prior automatic stage (Prior Scientific Instruments Ltd., Cambridge, UK). Image-Pro 6.2 with Stereologer plug-in software (MagWorldwide, Wokingham, UK) was used to select random fields for counting and to place a grid over the tissue. Germ cell counting used testis sections immunostained for VASA or OCT4. Germ cell counts in Pnd25 testes used methods described previously^59^,^60^. Relative cell volume per testis was determined by point counting^59^,^60^. The number of fields counted per animal (~10–15 fields) was dependent on obtaining a percentage SEM value of <5.

### Effects on fertility (F1)

Female adult rats that had been exposed in utero to indomethacin or acetaminophen were each placed with a control male for 4 days to allow mating, and the number of pups per litter counted on the day of birth. A similar mating procedure was followed for adult male F1 animals (n = 13 controls, n = 11 indomethacin-exposed, n = 5 acteminophen-exposed), each of which was placed with an untreated control female for 4 days and the number of pups determined at birth.

### Effects on the F2 generation

Animals exposed in utero (F1 generation) to indomethacin, acetaminophen or corn oil were mated as adults with opposite sex control animals and the resulting F2 offspring then studied at Pnd25 or in adulthood; the focus was primarily on female F2 offspring as male F2 offspring did not exhibit any obvious reproductive phenotypic change. Details of the numbers of F1 animals mated and the numbers of F2 offspring studied are given in the Results.

### Statistics

All presented data comparing the comparative effects of indomethacin or acetaminophen exposure was analysed using one-way ANOVA followed by Dunnett’s test. In the few instances in which comparison involved only two groups (vehicle v indomethacin), analysis was by Student’s t-test. All analyses used GraphPad Prism (version5; GraphPad Software Inc., San Diego, CA, USA). Where significant heterogeneity of variance was indicated, log transformation of data was undertaken to normalise variances prior to statistical analysis.

## Additional Information

**How to cite this article**: Dean, A. *et al*. Analgesic exposure in pregnant rats affects fetal germ cell development with inter-generational reproductive consequences. *Sci. Rep.* 6****, 19789; doi: 10.1038/srep19789 (2016).

## Supplementary Material

Supplementary Information
