# Supplementary material for: Analgesic exposure in pregnant rats affects fetal germ cell development with inter-generational reproductive consequences
Source: Sci Rep. 2016 Jan 27;6:19789. doi: 10.1038/srep19789 (PMC4728385; doi:10.1038/srep19789)
Supplement: Supplementary Information [file srep19789-s1.pdf]

Supplementary information for:

Analgesic exposure in pregnant rats affects fetal germ cell development with inter-generational reproductive consequences

Afshan Dean*[,1], Sander van den Driesche*, Yili Wang, Chris McKinnell, Sheila Macpherson, Sharon L Eddie[2], Hazel Kinnell, Pablo Hurtado-Gonzalez, Tom J Chambers, Kerrie Stevenson, Elke Wolfinger, Lenka Hrabalkova, Ana Calarrao, Rosey AL Bayne, Casper P Hagen[3], Rod T Mitchell, Richard A Anderson & Richard M Sharpe[4]

MRC Centre for Reproductive Health, The Queen's Medical Research Institute University of Edinburgh, 47 Little France Crescent, Edinburgh EH16 4TJ, UK

[1]Present address: Research Institute of Cardiovascular and Medical Sciences, University of Glasgow, Glasgow G12 8QQ

[2]Present address: Centre for Cancer Research and Cell Biology, Queen's University Belfast

[3]Present address: Department of Growth and Reproduction, Rigshospitalet, Faculty of Health and Medical Science, University of Copenhagen, Denmark

[4]To whom correspondence should be addressed

*Authors contributed equally to the manuscript

Running title: analgesics and fetal germ cell development

Keywords: Ovary, testis, indomethacin, acetaminophen (paracetamol), cyclooxygenase 2 (COX2), prostaglandin E_2 (PGE_2), PG-EP2/EP4 receptors, DMRT1, STRA8, LIN28, OCT4, VASA, anti-müllerian hormone (AMH)

Dean et al Supplementary information

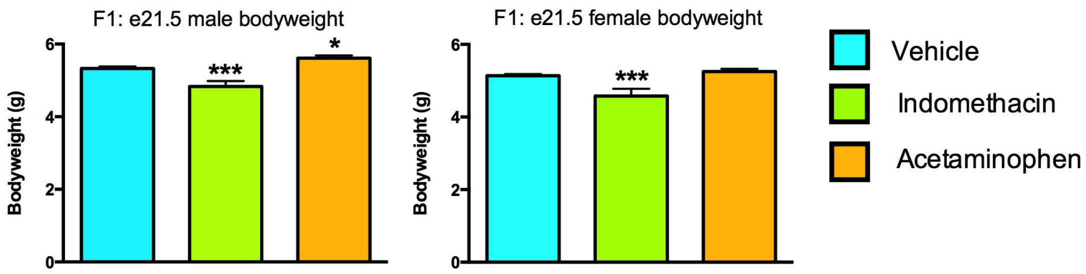

Supplementary Figure 1. Effect of fetal exposure to indomethacin or acetaminophen on F1 fetal bodyweight at e21.5. Values are Means ± SEM for n=21-72 animals per group. *p<0.05, **p<0.01, ***p<0.001, in comparison with respective control group. Animals derived from 4-12 litters per group. Analysis was by ANOVA followed by Dunnett's test.

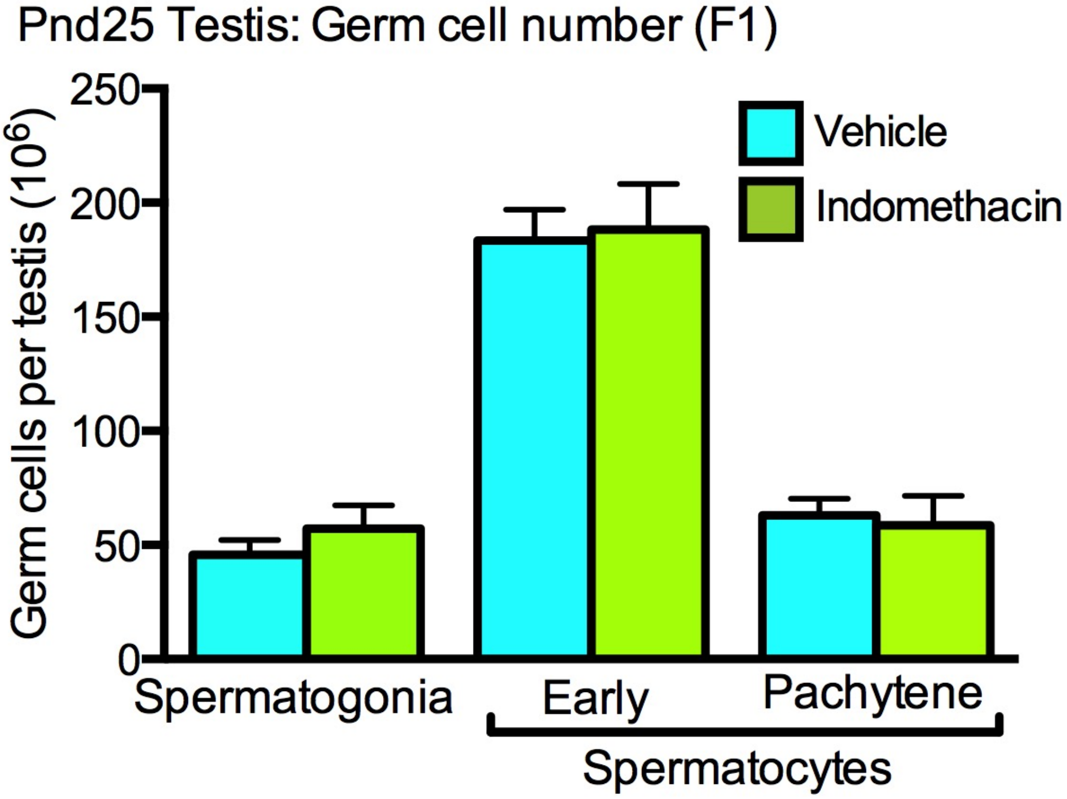

Supplementary Figure 2. Compensatory recovery of normal germ cell numbers by early puberty (Pnd25) in the testes of F1 rats exposed in utero to indomethacin (green bars). Data for corresponding controls (vehicle-exposed) are shown by the blue bars. Values are Means ± SEM for n=5-7 per group from 4 litters. Analysis was by Student's t test for each germ cell type.

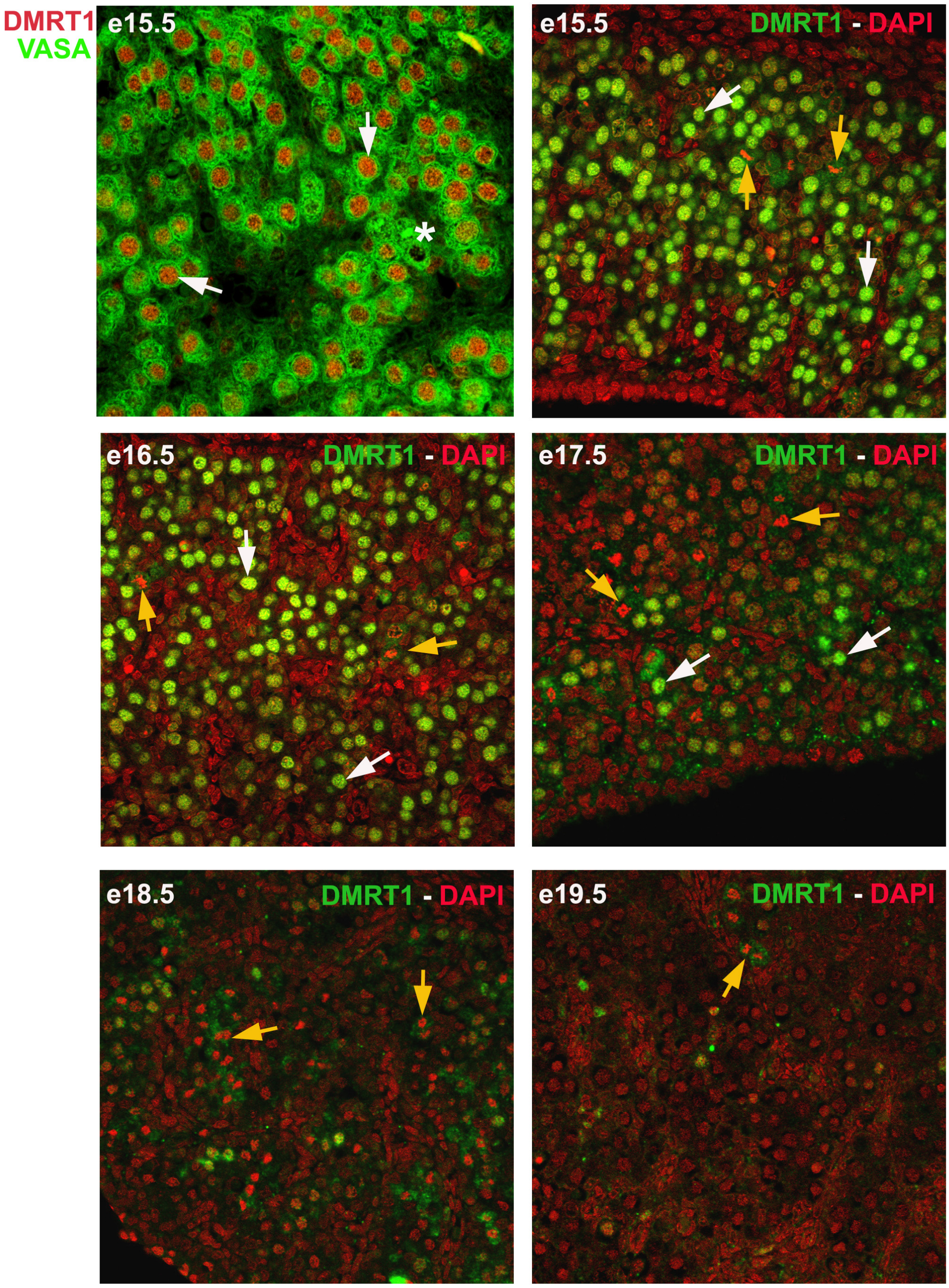

Supplementary Figure 3. Age-dependent change in germ cell-specific immunoexpression of DMRT1 in the fetal rat ovary. The top lefthand panel shows that from e15.5 onwards, expression of DMRT1 (red) is germ cell-specific (germ cells stained green for VASA; arrows); note that as germ cells enter meiosis they lose expression of DMRT1 (asterisk). The remaining panels show the temporal loss of germ cell expression of DMRT1 (green) between e15.5 and e19.5. White arrows show germ cells that have yet to enter meiosis, as indicated by nuclear DMRT1 expression. Yellow arrows show germ cells that have entered meiosis and in which DMRT1 expression has moved into the cytoplasm; after this stage, DMRT1 expression in germ cells is lost completely.

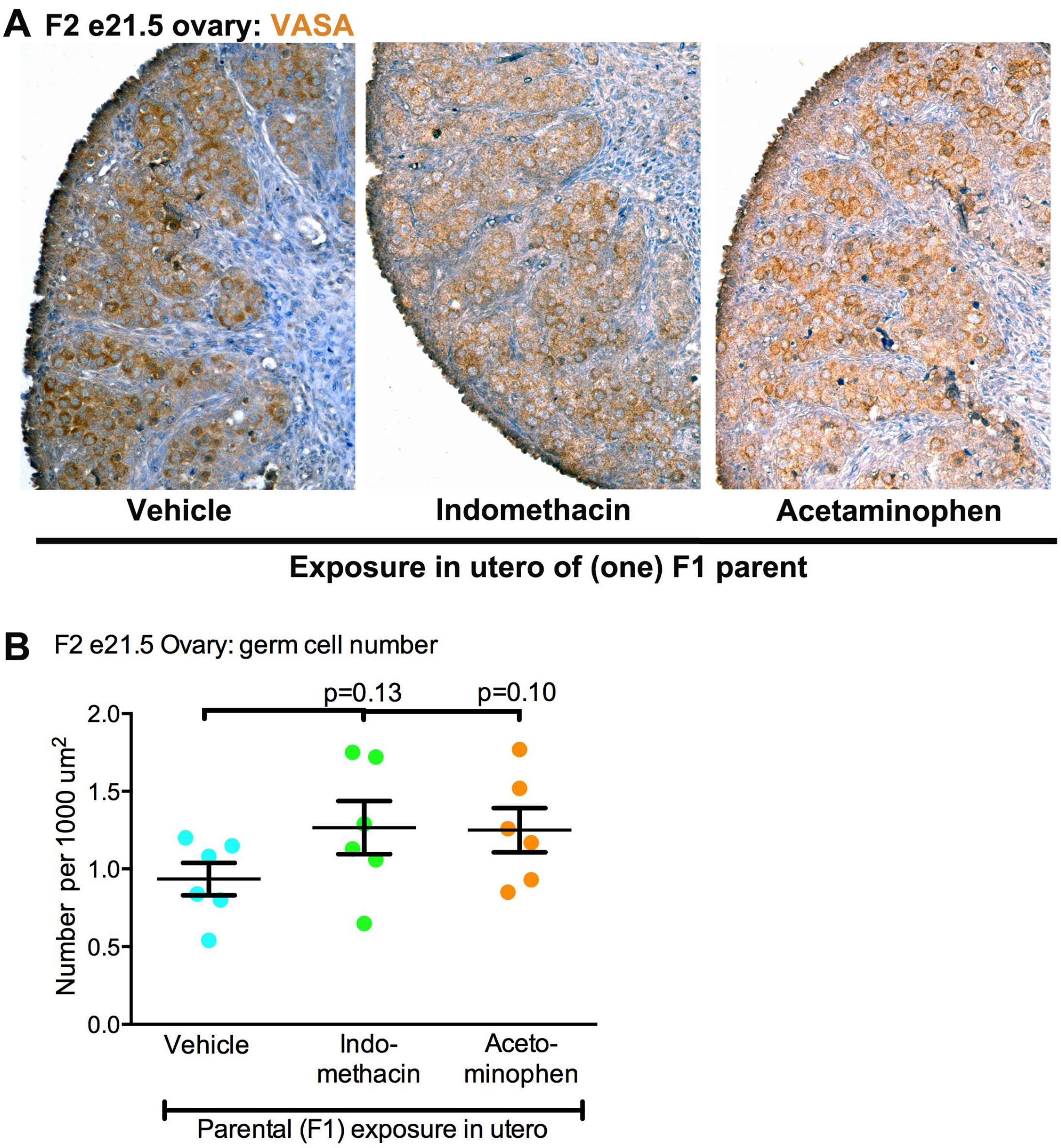

Supplementary Figure 4. Unchanged germ cell number in the fetal ovaries (e21.5) of F2 rats according to the in utero exposure of one or other F1 parent. **A**. Germ cell identification by cytoplasmic staining for VASA (brown). B. Germ cell quantification (Means $\pm$ SEM for n=6 fetuses from 5-6 separate litters), based on analysis of 3 cross-sections per ovary as outlined in Materials & Methods; Analysis was by ANOVA followed by Dunnett's test.

Supplementary Table 1. Details of antibodies used for immunohistochemistry.

**Primary antibody**	**Source**	**Antibody dilution**	**Secondary antibody (dilution)**
Antibodies used for colourimetric immunohistochemistry			
OCT4	Santa Cruz	1:40	RAG, biotin (1:500)
VASA	Abcam	1:40	SwAR, biotin (1:500)
YBX2	Thermo Scientific	1:200	GAR-biotin (1:500)
Antibodies used for fluorescence immunohistochemistry			
COX2	Santa Cruz	1:100	ChAR.P (1:200)
EP2	Cayman	1:2000	GAR-P (1:200)
DMRT1	Gift: Dr M Murphy, University of Minnesota	1:750	ChAR.P (1:200)
TRA98	Gift: Prof Y Nishimune, Osaka University	1:400	RARat.P (1:200)
VASA	Abcam	1:250	GARA.P (1:200)

RAG: rabbit anti-goat, SwAR: swine anti-rabbit, GAR: goat anti-rabbit, ChAR-P: chicken anti-rabbit peroxidase, ChAG-P: chicken anti-goat peroxidase, GACh-P: goat anti-chicken peroxidase, RARatP: rabbit anti-rat peroxidase, GAR-P: goat anti-rabbit peroxidase.

Supplementary Table 2. Primers used for gene expression quantification.

**Gene**	**Forward primer**	**Reverse primer (Roche probe no.)**
Cox2	cagcttcacttgccacca	ggttggaacagcaaggattt (74)
Ep2	cggacacccttacttctacagg	agaacagaagagcaaggagacc (121)
Dmrt1	cagaagccaaagcaagtgtg	agctgctggagagggaaac (129)
Stra8	ccagaatgtactctgagaatgaaagt	ttcctccttcttcagcatcc (46)
Lin28a	ccctagcaacgggttgtg	aagggctgggagtgaggt (62)
